# Mapping knowledge domain of acupuncture for Parkinson’s disease: a bibliometric and visual analysis

**DOI:** 10.3389/fnagi.2024.1388290

**Published:** 2024-09-04

**Authors:** Yanqing Zhao, Li Huang, Wentao Li

**Affiliations:** Shanghai Municipal Hospital of Traditional Chinese Medicine, Shanghai University of Traditional Chinese Medicine, Shanghai, China

**Keywords:** acupuncture, Parkinson’s disease, VOSviewer, CiteSpace, bibliometric analysis

## Abstract

**Objective:**

This study points to probing the inclination and mapping knowledge domain of acupuncture for Parkinson’s disease through bibliometrics.

**Methods:**

A search was conducted on 1 February 2024 using the Web of Science to identify papers published on acupuncture for Parkinson’s disease. The analysis included scientific research, countries, organizations, authors/cited authors, keywords, journals, and cited references. Bibliometric data were analyzed using VOSviewer software, CiteSpace, GraphPad Prism, and Scimago Graphica. The studies on acupuncture for Parkinson’s disease were visualized as a network map according to the publication year.

**Results:**

The cumulative publication trend on acupuncture for Parkinson’s disease is increasing year by year. China is the leading contributor in this field. International collaboration is predominantly concentrated in Europe, while institutional collaboration is chiefly limited to Chinese universities specializing in traditional Chinese medicine. Park HJ is the most prolific author, with “Movement Disorders” being the journal with the most publications. “Brain Research” is identified as a key journal, reflecting a focus on neuroscience. Kim SN is the most cited author, while Eisenberg DM is a prominent author in this field. Research topics such as mouse models, systematic reviews, and non-motor symptoms are frequently explored, with messenger RNA of substantia nigra emerging as a notable keyword in this field. Choi YG’s 2009 paper, published in the *Neuroscience Letters* journal, is a critical reference in this field. Key papers include Eisenberg DM’s 1998 study on randomized trials of acupuncture for non-motor symptoms of PD, as well as research focusing on the neuroinflammatory regulatory mechanisms of acupuncture for PD.

**Conclusion:**

The bibliometric analysis offers an exhaustive generality of the advancement and worldwide trends in acupuncture treatments for Parkinson’s disease, shedding light on potential avenues for prospective research.

## 1 Introduction

Parkinson’s disease is the most common severe movement disorder and the second most common neurodegenerative disease after Alzheimer’s disease, with 400 to 1,900 cases per 100,000 people worldwide (Pringsheim et al., 2014; Kulisevsky, 2022). As the population ages, the incidence of PD is increasing year by year, with its prevalence expected to double by 2040 (Kowal et al., 2013). In European and American countries, the prevalence of PD among individuals aged 60 and over is approximately 1%, and it exceeds 4% among those over 80 years old. In 2016, there were approximately 6.1 million PD patients worldwide (Samii et al., 2004). Data indicate that China has become the “largest country for Parkinson’s disease.” It is anticipated that by 2030, the number of Parkinson’s disease patients in China will surge to approximately 5 million. It is predicted that more than 50% of Parkinson’s disease patients in the world will come from China (Dorsey et al., 2007). The main pathological feature of PD is the degenerative loss of dopaminergic neurons in the substantia nigra (Dickson, 2018). Current research reckons that the onset of PD may be related to factors such as mitochondrial dysfunction, immune abnormalities, oxidative stress, and apoptosis. It is also associated with age, genetics, environment, unhealthy lifestyle, and psychological factors (Dauer and Przedborski, 2003; Das et al., 2011). Clinical manifestations are characterized by bradykinesia, resting tremor, myotonia, and postural and gait abnormalities (Poewe et al., 2017). In addition to motor symptoms, PD patients also have non-motor symptoms such as sensory symptoms, autonomic nervous system dysfunction, emotional problems, and sleep disorders (Chaudhuri et al., 2006).

The treatment of Parkinson’s disease should adhere to “dose titration” to avoid acute adverse drug reactions and strive to achieve the medication principle of “achieving satisfactory clinical effects with as small a dose as possible.” Acupuncture is a simple, convenient, cost-effective, and safe and effective treatment method. In recent years, there have been many high-quality clinical randomized controlled trials and systematic reviews/meta-analyses proving that acupuncture can effectively improve the symptoms of PD patients (Li et al., 2023; Fan et al., 2022; Pereira et al., 2022). Therefore, we will shift our perspective to acupuncture treatment.

Bibliometrics is an interdisciplinary field that employs mathematical and statistical methods to quantitatively analyze all knowledge carriers (Zhao et al., 2021). Through the analysis of countries, institutions, authors/cited authors, keywords, and cited references, we can identify research hotspots, frontiers, and domain content. The software utilized includes CiteSpace (Chen et al., 2023), VOSviewer (Huang et al., 2024), and HistCite (Ma et al., 2022). Earlier bibliometric studies on acupuncture for Parkinson’s disease encompass global trends in the Research and Development of Acupuncture Treatment on Parkinson’s Disease from 2000 to 2021: A Bibliometric Analysis (Li et al., 2022), Research progress of ferroptosis in Parkinson’s disease: a bibliometric and visual analysis (Lu et al., 2023), and Analysis of phenolic compounds in Parkinson’s disease: a bibliometric assessment of the 100 most cited papers (Perdigao et al., 2023), among which Li Xiaoping’s research also shows solicitude for the analysis of acupuncture in the treatment of Parkinson’s disease, and their research had several flaws. First, the search time was different from that of this study. Second, the search terms for acupuncture needed to be more comprehensive, and no topic was synonymous with the Mesh database in PubMed. Theme-word searches may lead to incomplete data retrieved, and their research needs to perform cluster analysis on related topics. Finally, there are no disambiguation statistics on related topic research. For example, in the analysis of countries, the United Kingdom is divided into four parts: England, Wales, Scotland, and Northern Ireland, of which England is only one part. This kind of statistics needs to be more accurate, and the keyword analysis has similar problems. Moreover, the latter two studies do not overlap with our study. Accordingly, we aim to offer valuable research insights for subsequent scholars. By integrating various bibliometric software tools, we have developed a pertinent knowledge map and identified the focal points and emerging trends within this domain, representing significant contributions to this study.

## 2 Methods

### 2.1 Source of literature

First, we acquired the synonyms for “Parkinson’s disease” and “acupuncture” through the MeSH database in PubMed and then amalgamated the final data. Next, we imported the Web of Science database with English Topic = “Parkinson’s disease,” chose “AND” as the logical relation word, and retrieved the literature literally. The search plan is as follows: TS = “Parkinson’s disease” AND “acupuncture”; TS = “Parkinson’s disease” AND “acupuncture therapy”; TS = “Parkinson’s disease” AND “Acupuncture, Ear”; TS = “Parkinson’s disease” AND “Acupuncture Points”; TS = “Parkinson’s disease” AND “Acupuncture Analgesia” separately (Huang et al., 2021). We will rely on the topic words “Parkinson,” “Parkinson’s disease,” and “Parkinsonism” with synonyms for “acupuncture” to install the above strategy and then search the database. Subsequently, configuration for the WoS database was carried out, with a search date set to 1 February 2024 and a date range from 1 January 1995 to 31 December 2023.

The Web of Science Core Collection is designed for researchers to find published literature. Web of Science Core Collection: Chemical Indexes, as well as SSCI, A&HCI, CPCI-S, CPCI-SSH, BKCI-S, and BKCI-SSH, are inappropriate for “Mapping Knowledge Domain of acupuncture for Parkinson’s disease: a bibliometric and visual analysis.” The language of the literature is not limited. Web of Science is the most trusted and independent global citation database in the world. The characteristics of Web of Science databases mainly include high-quality academic information, extensive subject coverage, strict screening mechanisms, unique citation indexing functions, and rich analysis tools (Zhang et al., 2024). It basically covers all the papers included in PubMed database. In addition, the Web of Science database has the following characteristics:

(1) High-quality academic information: The Web of Science Core Collection follows strict selection criteria, selecting the world’s most academically influential high-quality journals, and fully includes all information of each article, including comprehensive citation information. SCI, SSCI, and CPCI have always been recognized as the most authoritative scientific and technological literature indexing tools worldwide, providing the most important research results in the field of science and technology.
(2) Wide disciplinary coverage: Web of Science includes over 21,900 world-renowned and highly influential academic journals, covering fields such as natural sciences, engineering and technology, biomedical sciences, social sciences, arts, and humanities, dating back to 1900.
(3) Strict screening mechanism: The Web of Science Core Collection has a strict screening mechanism, based on Bradford’s Law in bibliometrics, only including important academic journals in various disciplines. The selection process is unbiased and has been tested for over half a century.
(4) Unique citation index function: The Web of Science database also includes the references cited in the paper and compiles a unique citation index based on the cited author, source, and publication year. Users can easily trace the origin and history of research literature or track its latest progress.
(5) Rich analysis tools: Web of Science provides various analysis tools, such as citation analysis, author analysis, and institutional analysis, to help users gain a deeper understanding of research trends and developments in a certain field.

In summary, Web of Science is a comprehensive, high-quality, and powerful academic literature retrieval platform that provides important information retrieval and academic communication tools for researchers. Based on this, this is the main basis for our literature search and analysis using this database. Moreover, most recent related studies (Dol et al., 2024; He et al., 2024; Xu et al., 2024) have also been based on the Web of Science database, demonstrating its authority in the field of bibliometric analysis. Then, these are the reasons why we use this database.

The citation index was set to “SCI-EXPANDED, SSCI,” and no language restrictions were applied. The next step involved deduplication to ensure the uniqueness of the literature. Initially, CiteSpace software was employed to eliminate duplicates. Subsequently, duplicate data were removed through a two-person data comparison process, ultimately yielding 281 articles. Two researchers verified the data both before and after the deduplication process. The researchers compared each paper individually, focusing on the title, abstract, and full text. We found that the consistency between the two researchers reached 100%, which suggested a strong correlation (Zhu et al., 2019). If there was any inconsistency, a third auditor was added and the data were compared again. The searched Web of Science database stems from China’s Tsing Hua University Library database.

### 2.2 Data analysis

The retrieved data were imported into CiteSpace 5.1.R8.SE (Dai et al., 2023) and VOSviewer 1.6.19 (Mamat et al., 2024), respectively. Subsequently, leveraging two bibliometric analysis tools, we separately constructed knowledge graphs for countries, institutions, authors, cited authors, keywords, journals, and cited references. In addition, GraphPad Prism facilitates the creation of trend graphs for scientific research outcomes. Then, the role of Scimago Graphica (Li et al., 2023) is to construct geographic visualization maps of scientific research output from various countries. Furthermore, CiteSpace provides the module value and silhouette value to appraise the effect of the atlas described, and *Q* > 0.3 indicates that the obtained community structure is significant. When *S* > 0.7, it indicates that the clustering result is the most reliable. If *S* > 0.5, clustering is usually reliable (Miao et al., 2017). Centrality is an important indicator, and the higher the centrality, the higher the correlation between the keyword and other keywords, belonging to the core of the domain (Zhu et al., 2022).

### 2.3 Parameter settings

When constructing a knowledge graph with Citespace, the specific settings include “pathfinder” and “pruning the merged network”. The year selection for “Years Per Slice” differs. For Cited Reference, Cited Author, and Author, the setting is “3”; for Keyword, it’s “8”; and for Cited Journal, it’s “6”. For VOSviewer analysis, the parameter settings are as follows: Method-LinLog; and graphs constructed-Network Visualization and Density Visualization. The specific analysis process mentioned above can be found in the analysis flowchart (Supplementary Figure S1).

## 3 Results

### 3.1 Analysis of the scientific research output

**Figure 1:**
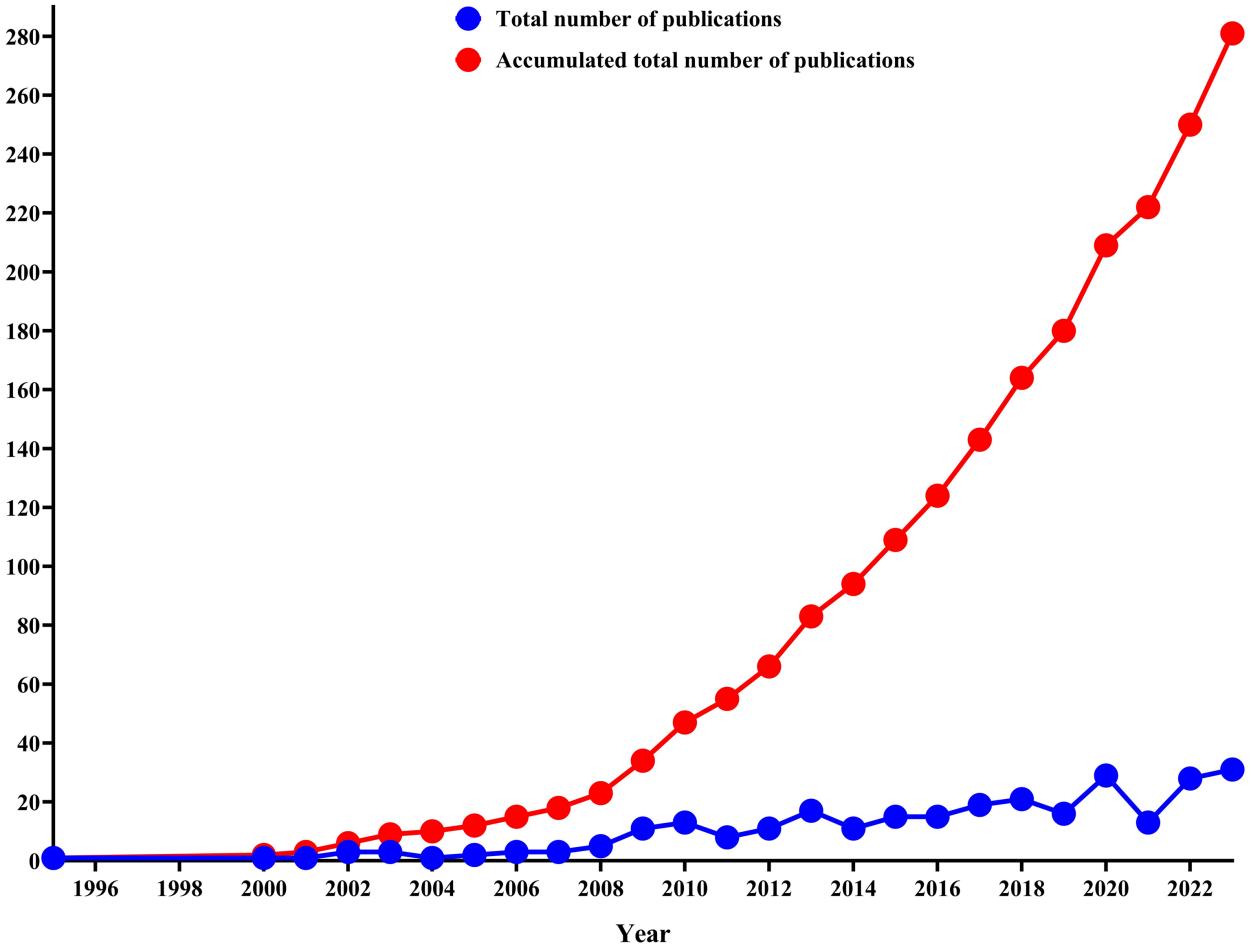
The total number of publications and accumulated total number of publications on acupuncture for Parkinson’s disease.

The earliest study in the domain can be retrospected back to 1995. Initially, one article was published, reaching 31 in 2023. As shown in Figure 1, from 1995 to 2007, the output of publications remained stable at a baseline level of growth. It can also be seen from the average number of publications, which fluctuates slightly around 2.0. From 2008 to 2014, the research output in this field demonstrated a substantial yearly increase, sustaining an average of approximately 10.85. This represented a notably significant growth trend compared to the period from 1993 to 2007. Subsequently, from 2015 to 2023, the growth trend stabilized once more, reaching an average research output of 20.77. However, there were some fluctuations in 2019 and 2021. The aforementioned development trend is further corroborated by the rising trajectory of the cumulative publication count, which demonstrates a consistent yearly increase.

### 3.2 Analysis of countries and institutions

**Figure 2:**
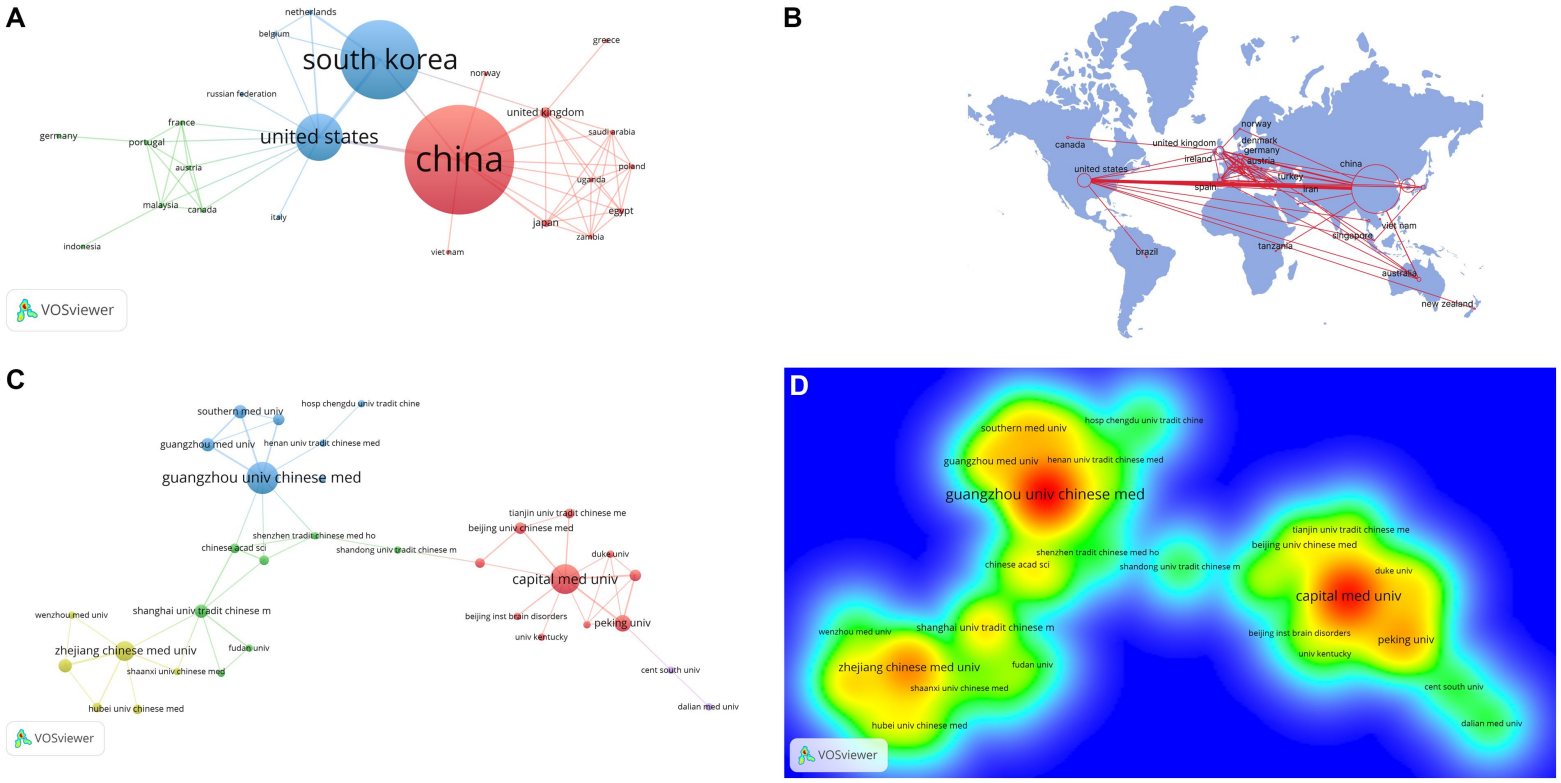
The visualization of countries **(A)**, geographical distribution **(B)**, institutions **(C)** and Rainbow map on acupuncture for Parkinson’s disease **(D)** (**A**: different nodes represent different countries, and the size of the nodes represents the number of publications; **B**: the color shades represent the number of published publications; **C**: different nodes represent different institutions, and the size of the nodes represents the number of publications; **D**: The darker the yellow, the higher the frequency of appearance).

The research cognate to this relates to 33 countries and 361 organizations, among which the top 5 countries with output are China, South Korea, USA, United Kingdom, and Japan, and the most cited countries are China (citation = 1856) and South Korea (citation = 1833) (Table 1). Figures 2A,B reveal the most robust cooperative relationships among China, South Korea, and the USA. From the cooperative relationship network perspective, most cooperative relationships are mainly concentrated between European countries, and there are more cooperation networks between European countries.

**Table 1:** Countries and organizations contributed to publications on acupuncture for Parkinson’s disease.

Country	Documents	Citations	Organization	Documents	Citations
China	123	1,856	Kyung hee univ	57	1,529
South Korea	86	1,833	Guangzhou univ. Chinese med	19	143
USA	48	1,768	Capital med univ	17	460
UK	9	248	Korea inst oriental med	15	237
Japan	6	88	Pusan natl univ	14	293
Egypt	5	120	China med univ	13	192
Netherlands	4	72	China med univ. hosp	12	163
France	3	443	Seoul natl univ	10	240
Germany	3	8	Zhejiang Chinese med univ	9	57
India	3	60	Kyung hee univ. hosp gangdong	7	200
Portugal	3	419	Peking univ	7	298
Singapore	3	87	Sang ji univ	7	98
Canada	2	418	Dongguk univ	6	130
Greece	2	11	Daejeon univ	5	39
Iran	2	48	Guangzhou med univ	5	38

In this table, the other two identical columns represent different rankings. The left column of the table represents the countries that have published acupuncture for Parkinson’s disease. The frequency of contribution is sorted from high to low.

Guangzhou Univ Chinese med, Guangzhou University of Chinese Medicine; Kyung hee univ, Kyung Hee University; Capital med univ, Capital Medical University; Korea inst oriental med, Korea Institute of Oriental Medicine; Pusan natl univ, Pusan National University; China med univ, China Medical University; China med univ hosp, Hospital of China Medical University; Seoul natl univ, Seoul National University; Zhejiang Chinese med univ, Zhejiang Chinese Medical University; Kyung hee univ hosp gangdong, Kyung Hee University Hospital at Gangdong; Peking univ, Peking University; Sang ji univ, Sang-ji University; Dongguk univ, Dongguk University; Daejeon univ, Daejeon University; Guangzhou med univ, Guangzhou Medical University.

Table 1 presents the institutions that are ranked within the top five in terms of scientific research output, specifically Kyunghee University, Guangzhou University of Chinese Medicine, Capital Medical University, Korea Institute of Oriental Medicine, and Pusan National University, and Kyung Hee University (citation = 1,529) has the most cited output. In addition to validating the rules as mentioned above, Figures 2C,D reveal a distinctive characteristic of institutional cooperation networks: They are primarily centered in Beijing, Zhejiang, and Guangzhou, respectively, exhibiting a north–south distribution pattern within China. Moreover, the robust network connections among these institutions are confined predominantly within China. Furthermore, the network relationships among these institutions are largely restricted to China’s more developed first-tier cities, with a notable absence of cooperation with institutions from other countries—an area that requires enhancement in the future.

### 3.3 Analysis of authors

**Figure 3:**
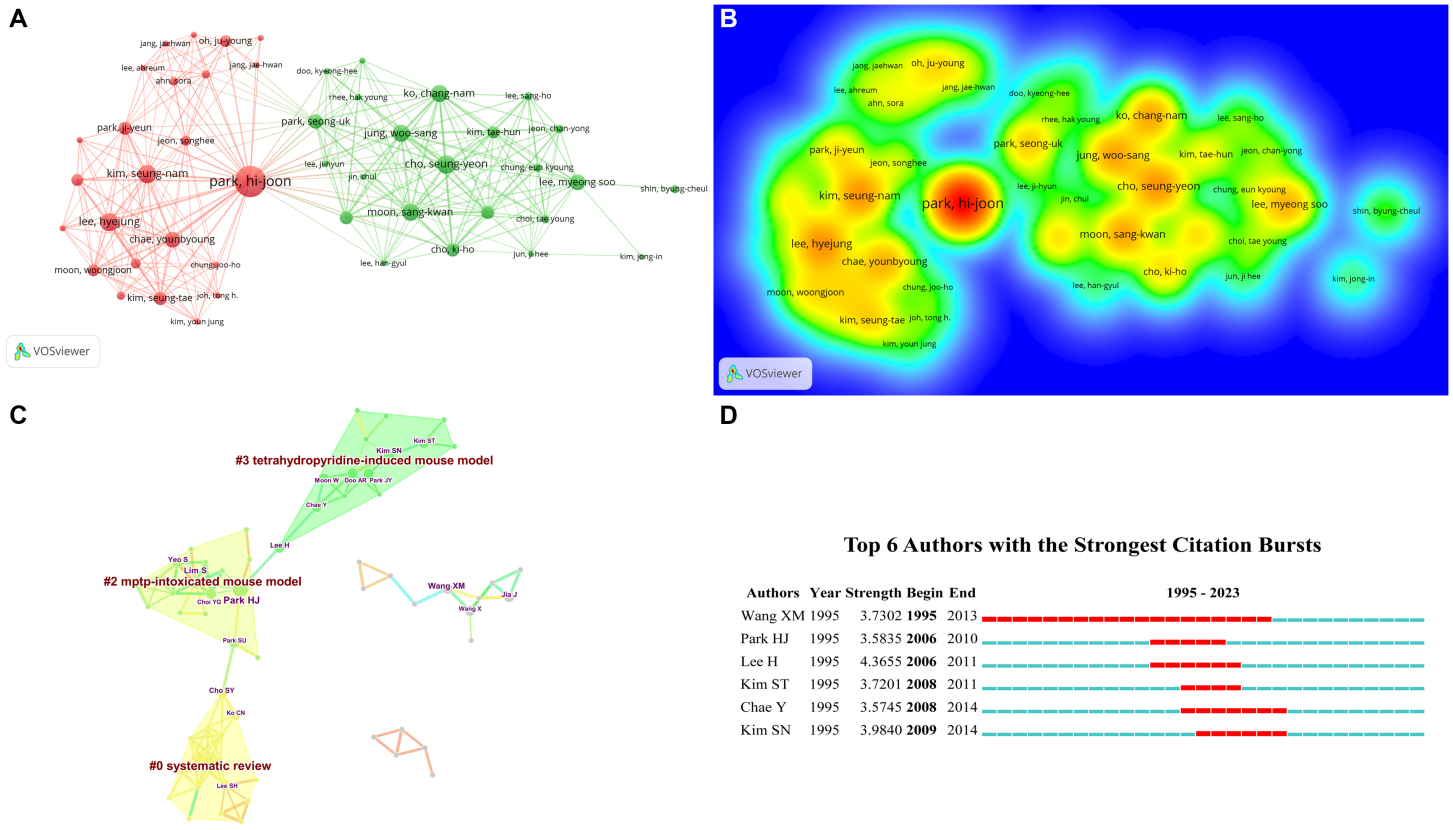
The visualization of authors **(A)**, Rainbow map **(B)**, and Cluster map **(C)** of authors based on label clusters with title terms, and Authors with the Strongest Citation Bursts **(D)** (**A**: different nodes represent different authors, and the size of the nodes represents the number of publications; **B**: The darker the yellow, the higher the frequency of appearance; **C**: Different color blocks represent different clustering categories; **D**: The red bar represents the persistence of the author’s popularity).

An analysis of reported document and paper citations reveals that the top 10 authors, ranked by the number of papers published, are presented in Table 2. Park HJ, with 23 papers, leads the list as the most productive author in this research area, followed by Lim S, Yeo SJ, Jia J, and Cho SY (Figures 3A,B). Moreover, Park HJ is also the author with the most citations; they are the most prominent researchers in the author’s network relationship.

**Table 2:** Authors, cited authors, keywords, and cited references contributed to publications on acupuncture for Parkinson’s disease.

No.	Author	Frequency	Citations	Cited author	Citations	Total link strength	Cited reference	Frequency	Cited reference	Centrality	Keyword	Frequency
1	Park, Hi-joon	23	802	Kim, SN	79	1,826	Cho SY (2012)	41	Choi YG (2009)	0.61	Acupuncture	195
2	Lim, Sabina	15	366	Yeo, S	76	1,961	Jeon S (2008)	31	Choi YG (2011)	0.51	Parkinson’s disease	162
3	Yeo, Sujung	13	218	Cho, SY	74	1,454	Kang JM (2007)	29	Doo KH (2015)	0.42	Stimulation	31
4	Jia, Jun	11	288	Liang, XB	74	1,524	Kluger BM (2016)	28	Kang JM (2007)	0.37	Mouse model	26
5	Cho, Seung-yeon	10	216	Shulman, LM	65	1,177	Zeng BY (2016)	27	Cho SY (2018)	0.33	Messenger-rna	25
6	Kim, Seung-nam	10	266	Park, HJ	58	1,131	Kim SN (2011)	26	Rajendran PR (2001)	0.33	Brain	24
7	Lee, Hyejung	10	445	Chae, Y	54	1,111	Doo KH (2015)	22	Park HJ (2003)	0.31	Substantia-nigra	24
8	Jung, Woo-sang	9	216	Choi, YG	47	999	Chae Y (2014)	22	Eisenberg DM (1998)	0.30	Medicine	22
9	Ko, Chang-nam	9	216	Kang, JM	47	917	Kim SN (2009)	22	Lv E (2015)	0.30	Systematic review	21
10	Moon, Sang-kwan	9	216	Rajendran, PR	47	752	Wang HM (2011)	21	Lei H (2016)	0.29	Nonmotor symptoms	20

In this table, the other two identical columns represent different rankings. The left column of the table represents the authors who have published acupuncture for Parkinson’s disease. The frequency of contribution is sorted from high to low.

By rerunning the CiteSpace software, Figure 3C illustrates the clustering analysis, and ultimately, three cluster words are gained, as presented in Table 3: #0 systematic review, #2 MPTP-intoxicated mouse model, #3 tetrahydropyridine-induced mouse model. Through the Authors with the Strongest Citation Bursts, it can be seen that Wang XM is a famous researcher in this field (Figure 3D). Thus, it can be seen that scholars often use acupuncture therapy to conduct relevant research on Parkinson’s disease mouse models.

**Table 3:** Authors engaged in acupuncture for Parkinson’s disease that details of knowledge clusters.

Cluster ID	Size	Silhouette	Mean (year)	Label (LLR)	Label (MI)
0	18	0.97	2016	Systematic review	Systematic review (0.22)
2	17	0.942	2013	MPTP-intoxicated mouse model	Injecting brain cells (0.21)
3	14	0.945	2010	Tetrahydropyridine-induced mouse model	Injecting brain cells (0.35)

The cluster analysis results mainly include cluster ID, mean year, size, silhouette, label (LLR), and label (MI). Cluster ID is the number after clustering, and size represents the number of members contained in the cluster. The larger the size is, the smaller the number. The mean year represents the average year of the literature in the cluster, which can be used to judge the distance of the cited literature in the cluster. The larger the log-likelihood ratio (LLR), the more representative the cluster category; mutual information (MI) is mainly used to represent the relationship between terms and categories in text mining, and it does not consider the frequency of feature words.

### 3.4 Analysis of cited authors

**Figure 4:**
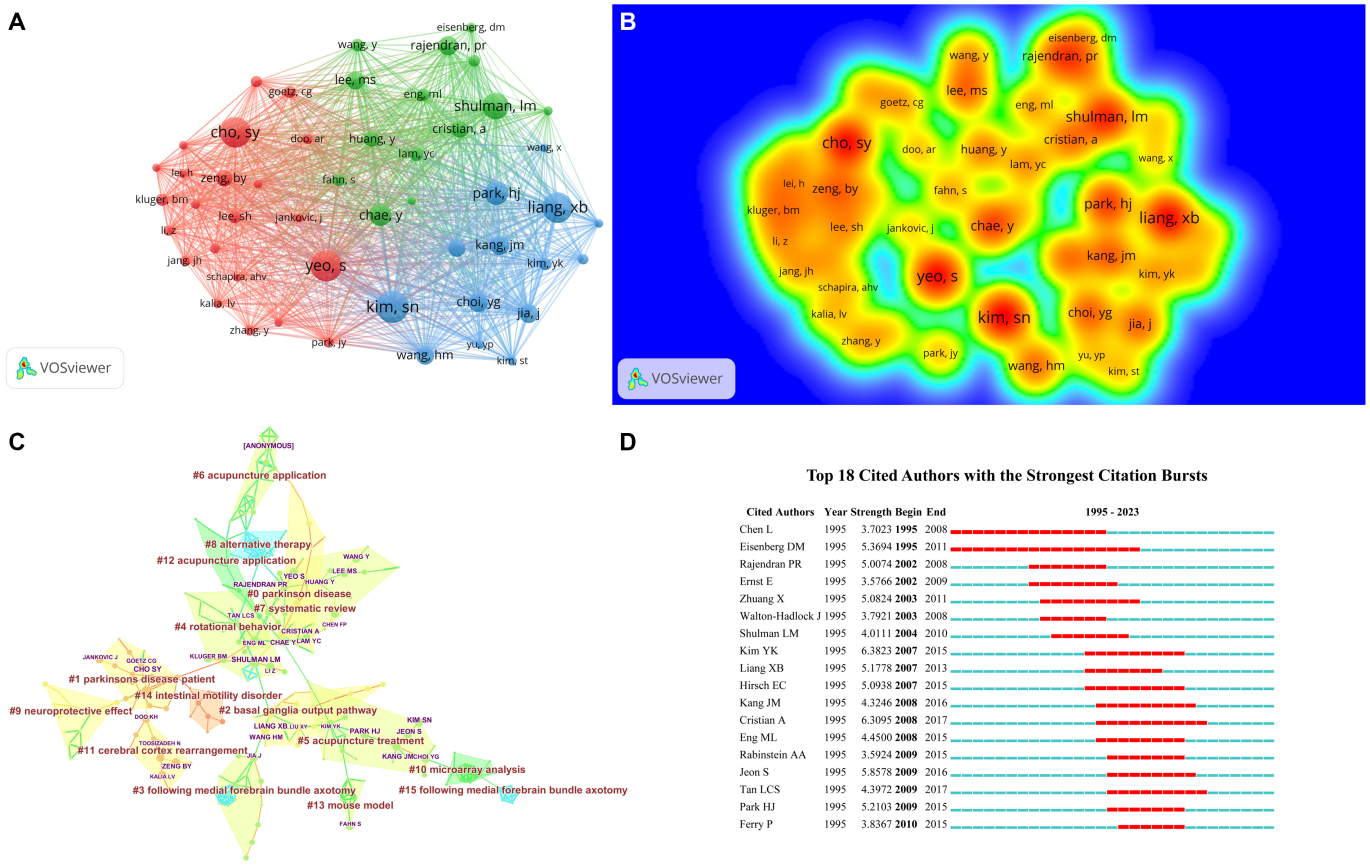
The visualization of cited authors **(A)**, Rainbow map **(B)**, Cluster map **(C)**, and Citation Bursts **(D)** of cited authors based on label clusters with title terms (**A**: different nodes represent different cited authors, and the size of the nodes represents the number of publications; **B**: The darker the yellow, the higher the frequency of appearance; **C**: Different color blocks represent different clustering categories; **D**:The red bar represents frequent citations, while the green bar represents infrequent citations).

Cited author refers to the phenomenon where other literature collectively cites two authors. When CiteSpace software calculates the co-citation of authors, it only counts the co-citation of the first author (Gao et al., 2023). By calculating the relationships among co-cited authors, we can create a co-citation network diagram, revealing the citation relationships of academic achievements within specific research fields. Table 2 shows that seven authors have received over 50 co-citations each. The author with the most citations is Kim SN (79 citations), closely followed by Yeo S and Cho SY. In addition, Figures 4A,B show the co-citation network diagram for 253 authors whose co-citations exceed 16. It also demonstrates the positive collaborative relationships among authors with different co-citations, such as the collaborations between Cho SY and Yeo S, Kim SN, Liang XB, and Shulman LM. Table 4 and Figure 4C present the clustering analysis results of the cited authors from running CiteSpace software, yielding 16 clusters: #0 Parkinson’s disease, #1 Parkinson’s disease patient, #2 basal ganglia output pathway, #3 following medial forebrain bundle axotomy, #4 rotational behavior, #5 acupuncture treatment, #6 acupuncture application, #7 systematic review, #8 alternative therapy, #9 neuroprotective effect, #10 microarray analysis, #11 cerebral cortex rearrangement, #12 acupuncture application, #13 mouse model, #14 intestinal motility disorder, #15 following medial forebrain bundle axotomy. The most substantial citation burst detection is utilized to observe study plumes within a specific time node. The “Citation/Frequency Burst History” feature in CiteSpace software is utilized to identify research hotspots within this domain. The red rectangular bars signify the duration of citation hotspots and denote the start and end years of citation peaks (Zhao et al., 2021). Upon examining Figure 4D, it is evident that the prominent author in this field has become Eisenberg DM.

**Table 4:** Cited authors engaged in acupuncture for Parkinson’s disease that details of knowledge clusters.

Cluster ID	Size	Silhouette	Mean (year)	Label (LLR)	Label (MI)
0	28	0.93	2011	Parkinson’s disease	d2 receptor level (1.07)
1	20	0.934	2018	Parkinson’s disease patient	d2 receptor level (0.68)
2	20	0.976	2014	Basal ganglia output pathway	d2 receptor level (0.53)
3	18	0.985	2009	Following medial forebrain bundle axotomy	d2 receptor level (0.43)
4	18	0.895	2009	Rotational behavior	d2 receptor level (0.1)
5	18	0.89	2009	Acupuncture treatment	d2 receptor level (0.51)
6	15	0.846	2008	Acupuncture application	Parkinson’s disease (0.08)
7	14	0.917	2014	Systematic review	d2 receptor level (0.19)
8	13	0.957	2002	Alternative therapy	Parkinson’s disease (0.11)
9	12	0.965	2013	Neuroprotective effect	d2 receptor level (0.22)
10	11	0.979	2011	Microarray analysis	Parkinson’s disease (0.07)
11	10	0.974	2018	Cerebral cortex rearrangement	d2 receptor level (0.22)
12	8	0.918	2007	Acupuncture application	Parkinson’s disease (0.12)
13	8	0.991	2010	Mouse model	Parkinson’s disease (0.09)
14	8	0.947	2022	Intestinal motility disorder	Parkinson’s disease (0.08)
15	5	0.989	2002	Following medial forebrain bundle axotomy	Parkinson’s disease (0.12)

The cluster analysis results mainly include cluster ID, mean year, size, silhouette, label (LLR), and label (MI). Cluster ID is the number after clustering, and size represents the number of members contained in the cluster. The larger the size is, the smaller the number. The mean year represents the average year of the literature in the cluster, which can be used to judge the distance of the cited literature in the cluster. The larger the log-likelihood ratio (LLR), the more representative the cluster category; mutual information (MI) is mainly used to represent the relationship between terms and categories in text mining, and it does not consider the frequency of feature words.

### 3.5 Analysis of journals

**Figure 5:**
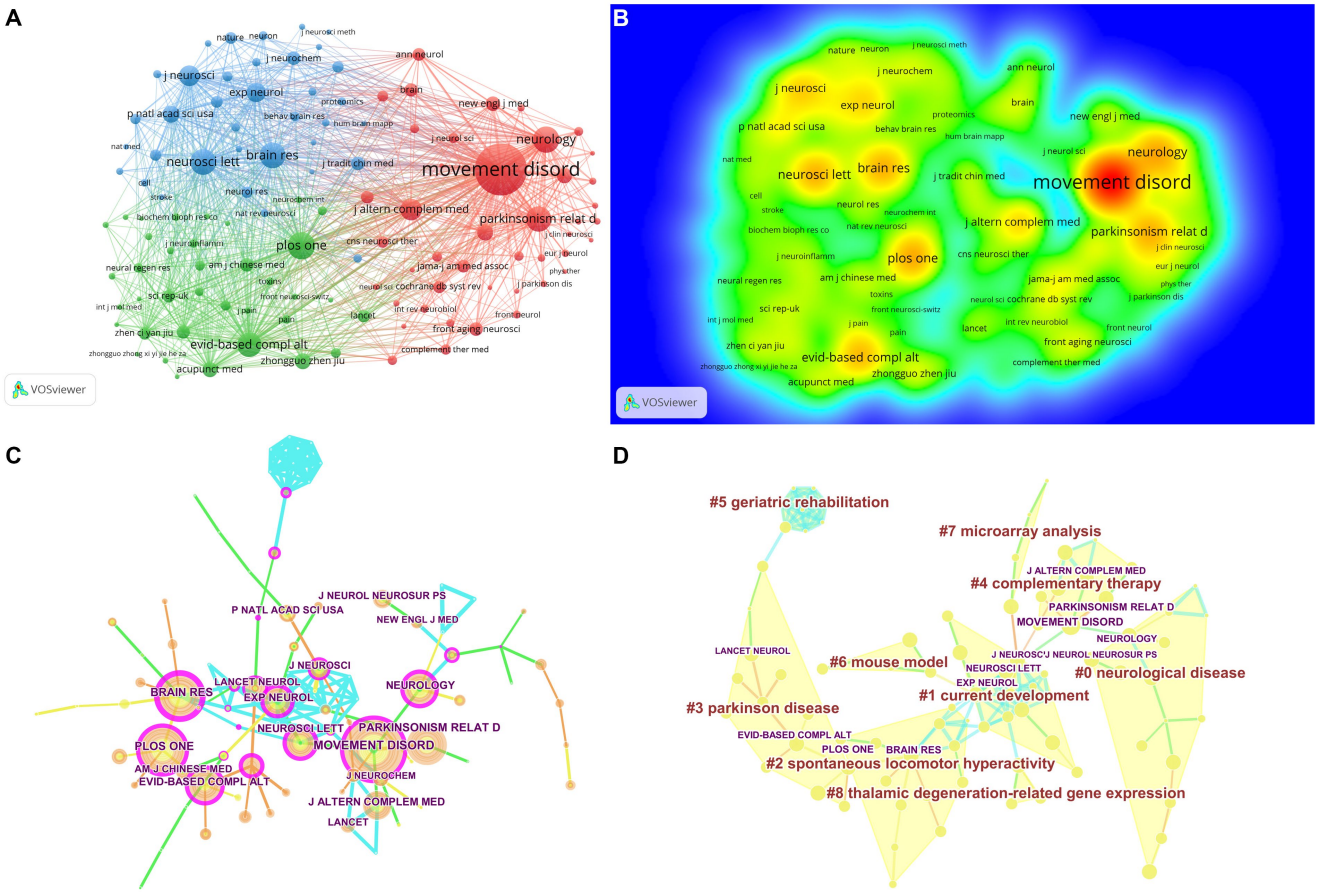
Network of journals **(A)**, Rainbow map **(B)**, Network of journals on acupuncture for Parkinson’s disease **(C)**, and Cluster map **(D)** of authors based on label clusters with title terms on acupuncture for Parkinson’s disease (**A**: different nodes represent different journals, and the size of the nodes represents the number of publications; **B**: The darker the yellow, the higher the frequency of appearance; **C**: Network of journals on acupuncture for Parkinson’s disease. The purple node in the middle of the annual ring means the influence and the significance of a journal. The larger the node and the more purple it exhibits, the greater is the importance of the journal; **D**: Different color blocks represent different clustering categories).

The distribution of the pivotal journals undertaken in acupuncture for Parkinson’s disease is revealed in Table 5. The most prolific journal was Movement Disorders (Frequency = 186), followed by Brain Research (Frequency = 137), Plos One (Frequency = 130), Parkinsonism & Related Disorders (Frequency = 128), and Neurology (Frequency = 115), and the most critical journal was Brain Research (centrality = 0.74), followed by Movement Disorders (centrality = 0.57), Neuroscience Letters (centrality = 0.55), Plos One (centrality = 0.54), and Experimental Neurology (centrality = 0.54) (Figures 5A–C). The top 10 journals publishing the most research on acupuncture for PD have an average impact factor of 4.805, with over half exceeding an impact factor of 3. Consequently, these journals are deemed pivotal in co-citation networks, indicating from another viewpoint that acupuncture and moxibustion are gaining increasing popularity.

**Table 5:** Journals contributed to publications on acupuncture for Parkinson’s disease.

Journal	Frequency	IF (2022)	Journal	Centrality	IF (2022)
Movement Disorders	186	8.60	Brain Research	0.74	2.90
Brain Research	137	2.90	Movement Disorders	0.57	8.60
Plos One	130	3.70	Neuroscience Letters	0.55	2.50
Parkinsonism & Related Disorders	128	4.10	Plos One	0.54	3.70
Neurology	115	10.10	Experimental Neurology	0.54	5.30
Journal of Alternative and Complementary Medicine	114	2.60	Evidence-based Complementary and Alternative medicine	0.48	2.65
Neuroscience Letters	107	2.50	Neurology	0.42	10.10
Evidence-based Complementary and Alternative medicine	101	2.65	Frontiers in Aging Neuroscience	0.42	4.80
Experimental Neurology	93	5.30	Lancet Neurology	0.30	48.00
Journal of Neurology	82	5.60	Acupuncture & Electro-therapeutics Research	0.29	0.30

In this table, the other two identical columns represent different rankings. The left column of the table represents the journals that have published acupuncture for Parkinson’s disease. The frequency of contribution is sorted from high to low. The column on the right represents the sorting from high to low according to centrality.

Figures 5C,D present the journal relationship graph and nine journal cluster maps generated by CiteSpace software (Table 6). The relationship graph comprises 101 nodes and 138 links. With a modularity *Q* value exceeding 0.7 and a silhouette value of 0.4001, it suggests the reliability of the identified community structure. These graphs, along with the cluster analysis maps, serve as valuable guides for future researchers to swiftly identify key journals within the field and understand the relationships between them as shown below: #0 neurological disease, #1 current development, #2 spontaneous locomotor hyperactivity, #3 Parkinson’s disease, #4 complementary therapy, #5 geriatric rehabilitation, #6 mouse model, #7 microarray analysis, #8 thalamic degeneration-related gene expression.

**Table 6:** Cited journal engaged in acupuncture for Parkinson’s disease that details knowledge clusters.

Cluster ID	Size	Silhouette	Mean (year)	Label (LLR)	Label (MI)
0	15	0.959	2006	Neurological disease	Striatal degeneration-related gene expression (0.54)
1	15	0.806	2003	Current development	Striatal degeneration-related gene expression (0.85)
2	14	0.844	2009	Spontaneous locomotor hyperactivity	Striatal degeneration-related gene expression (0.56)
3	13	0.848	2012	Parkinson’s disease	Striatal degeneration-related gene expression (2.43)
4	9	0.909	2006	Complementary therapy	Alternative medicine use (0.39)
5	8	0.989	2002	Geriatric rehabilitation	Parkinson’s disease (0.18)
6	5	0.966	2009	Mouse model	Parkinson’s disease (0.16)
7	4	0.924	2007	Microarray analysis	Parkinson’s disease (0.17)
8	3	0.957	2004	Thalamic degeneration-related gene expression	Parkinson’s disease (0.19)

The cluster analysis results mainly include cluster ID, mean year, size, silhouette, label (LLR), and label (MI). Cluster ID is the number after clustering, and size represents the number of members contained in the cluster. The larger the size is, the smaller the number. The mean year represents the average year of the literature in the cluster, which can be used to judge the distance of the cited literature in the cluster. The larger the log-likelihood ratio (LLR), the more representative the cluster category; mutual information (MI) is mainly used to represent the relationship between terms and categories in text mining, and it does not consider the frequency of feature words.

### 3.6 Analysis of keywords

**Figure 6:**
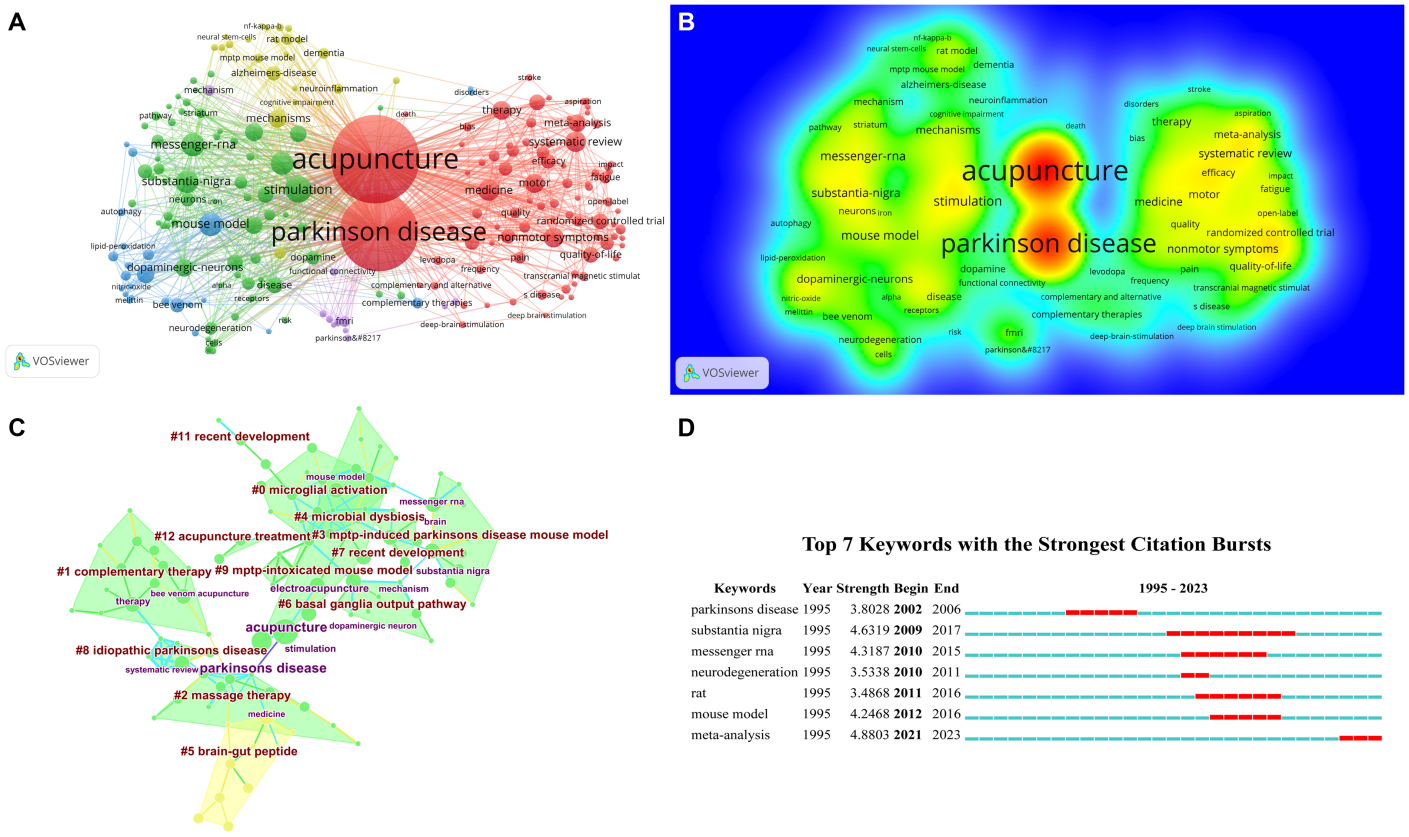
The visualization of keywords **(A)**, Rainbow map **(B)**, Cluster map **(C)**, and Citation Bursts **(D)** of keywords based on label clusters with title terms. (A: different nodes represent different keywords, and the size of the nodes represents the number of frequencies; B: The darker the yellow, the higher the frequency of appearance; C: Different color blocks represent different clustering categories; D: The red bar represents frequent citations, while the green bar represents infrequent citations).

Through the co-occurrence analysis of keywords, we can identify key topics and emerging trends in acupuncture for PD. In the study of acupuncture for Parkinson’s disease, 10 keywords appear more than 20 times, as displayed in Table 2 and Figure 6A, representing the primary research areas in this field. Excluding the keywords acupuncture and Parkinson’s disease, the research on acupuncture for Parkinson’s disease primarily focuses on non-motor symptoms, messenger RNA, and a systematic review of randomized controlled trials (Figure 6B).

Twelve clusters were derived from software-based cluster analysis (as depicted in Figure 6C and Table 7), with a modularity *Q* value of 0.7538. The mean silhouette value is 0.7367, which is greater than 0.7. The cluster visualization illustrates co-occurring author keywords and keyword plus, which comprised #0 microglial activation, #1 complementary therapy, #2 massage therapy, #3 MPTP-induced Parkinson’s disease mouse model, #4 microbial dysbiosis, #5 brain-gut peptide, #6 basal ganglia output pathway, #7 recent development, #8 idiopathic Parkinson’s disease, #9 MPTP-intoxicated mouse model, #11 recent development, #12 acupuncture treatment. Subsequently, using the burst analysis feature within the software, we identified the research hotspots associated with key terms in the field of acupuncture for PD, as depicted in Figure 6D. The research indicates that the primary focus of this field is on messenger RNA of substantia nigra.

**Table 7:** Keywords related to acupuncture for Parkinson’s disease that details knowledge clusters.

Cluster ID	Size	Silhouette	Mean (year)	Label (LLR)	Label (MI)
0	16	0.655	2008	Microglial activation	MPTP-intoxicated mouse model (0.25)
1	14	0.875	2013	Complementary therapy	MPTP-intoxicated mouse model (0.75)
2	12	0.891	2009	Massage therapy	MPTP-intoxicated mouse model (0.25)
3	11	0.942	2012	MPTP-induced Parkinson’s disease mouse model	MPTP-intoxicated mouse model (0.15)
4	11	0.876	2009	Microbial dysbiosis	Electroacupuncture-regulated neurotrophic factor miRNA expression (1.4)
5	10	0.885	2015	Brain-gut peptide	MPTP-intoxicated mouse model (0.15)
6	8	0.949	2010	Basal ganglia output pathway	Parkinson’s disease (0.05)
7	6	0.929	2006	Recent development	Parkinson’s disease (0.06)
8	6	0.965	2010	Idiopathic Parkinson’s disease	Idiopathic Parkinson’s disease (0.06)
9	4	0.959	2011	MPTP-intoxicated mouse model	Parkinson’s disease (0.06)
11	3	0.938	2009	Recent development	Parkinson’s disease (0.08)
12	3	0.923	2010	Acupuncture treatment	Parkinson’s disease (0.07)

The cluster analysis results mainly include cluster ID, mean year, size, silhouette, label (LLR), and label (MI). Cluster ID is the number after clustering, and size represents the number of members contained in the cluster. The larger the size is, the smaller the number. The mean year represents the average year of the literature in the cluster, which can be used to judge the distance of the cited literature in the cluster. The larger the log-likelihood ratio (LLR), the more representative the cluster category; mutual information (MI) is mainly used to represent the relationship between terms and categories in text mining, and it does not consider the frequency of feature words.

### 3.7 Analysis of cited reference

**Figure 7:**
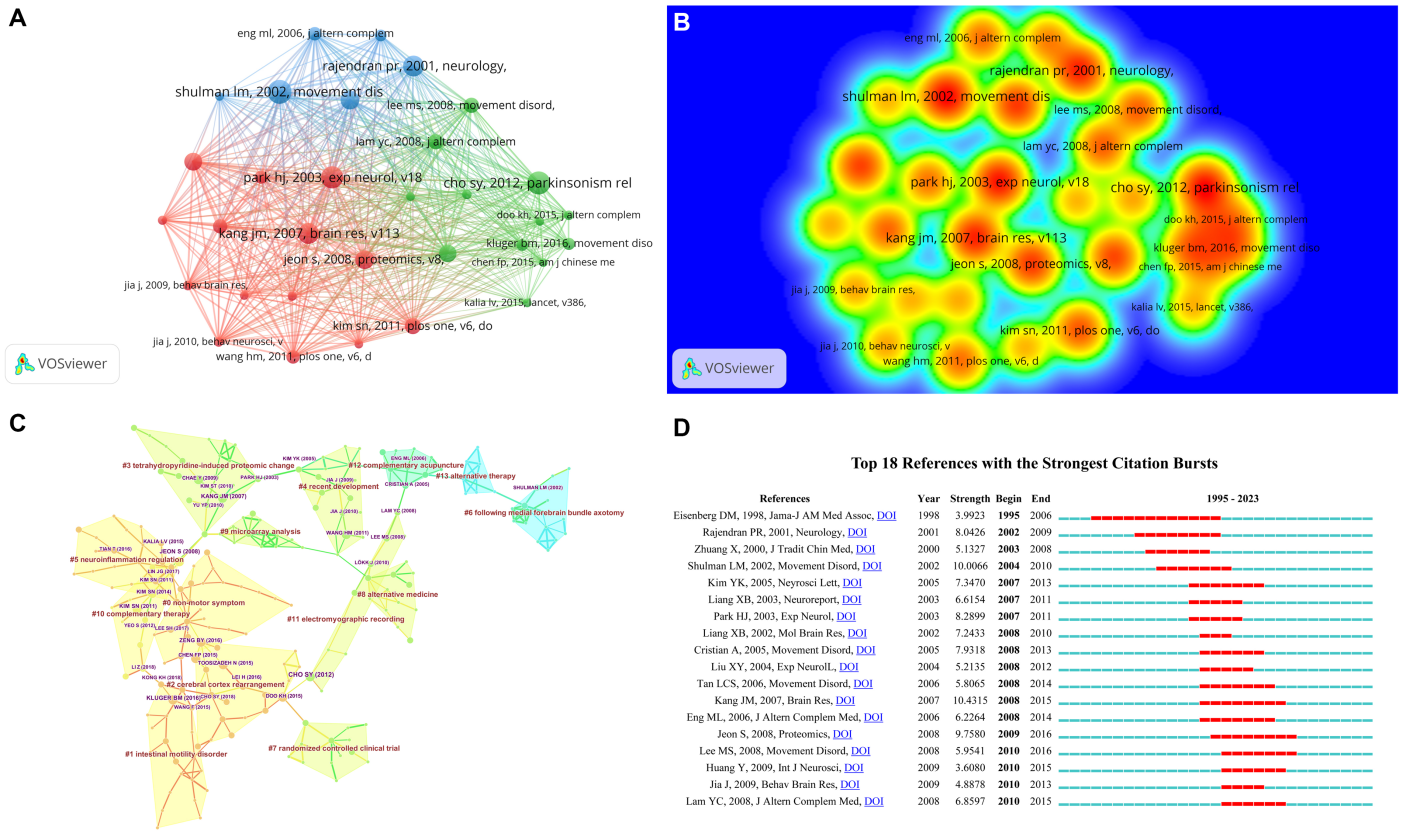
The visualization of cited reference **(A)**, Rainbow map **(B)**, Cluster map **(C)**, and Citation Bursts **(D)** of cited reference based on label clusters with title terms.

Cited references refer to the phenomenon where the same reference cites two references. The intellectual structure of a specific study domain can be revealed by analyzing the clustering and critical nodes in the co-citation network. Table 2 illustrates the distribution pattern of references, ranked by frequency and centrality. Moreover, Figures 7A,B display the top 5 references with the highest number of co-citations, specifically Cho et al. (2012) (Frequency = 41), Jeon et al. (2008) (Frequency = 31), Kang et al. (2007) (Frequency = 29), Kluger et al. (2016) (Frequency = 28), and Zeng and Zhao (2016) (Frequency = 27), and the highest centrality is Choi et al. (2009) (Centrality = 0.61) and is regarded as the most noteworthy reference in the field, followed by Choi et al. (2011) (Centrality = 0.51), Doo et al. (2015) (Centrality = 0.42), Kang et al. (2007) (Centrality = 0.37), and Cho et al. (2018) (Centrality = 0.33).

The results of the clustering analysis of the cited references, displayed in Figure 7C, exhibit a modularity *Q* value of 0.8611 > 0.7, signifying the credibility of the identified community structure. Table 8 presents the outcomes of the clustering analysis, encompassing the 14 largest clusters, which included #0 non-motor symptom, #1 intestinal motility disorder, #2 cerebral cortex rearrangement, #3 tetrahydropyridine-induced proteomic change, #4 recent development, #5 neuroinflammation regulation, #6 following medial forebrain bundle axotomy, #7 randomized controlled clinical trial, #8 alternative medicine, #9 microarray analysis, #10 complementary therapy, #11electromyographic recording, #12 complementary acupuncture, #13 alternative therapy. The clustering analysis of cited references indicates that research in the field encompasses non-motor symptoms of Parkinson’s disease and associated randomized controlled studies. In our research, Figure 7D illustrates the hotspot analysis of 18 cited references from the Burst History analysis, where the red bars represent the bursts in citations. Observations reveal that a highly cited study by Eisenberg DM, published in 1998 in the Journal of the American Medical Association, explored trends in alternative medicine use in the United States from 1990 to 1997. This nationwide follow-up survey suggested that acupuncture treatment for PD may be effective.

**Table 8:** Cited reference concerned with acupuncture for Parkinson’s disease that details knowledge clusters.

Cluster ID	Size	Silhouette	Mean (year)	Label (LLR)	Label (MI)
0	23	0.951	2018	Non-motor symptom	Bibliometric analysis (0.85)
1	21	0.91	2020	Intestinal motility disorder	Mice model (0.37)
2	21	0.909	2016	Cerebral cortex rearrangement	Mice model (0.94)
3	20	0.893	2008	Tetrahydropyridine-induced proteomic change	Mice model (0.24)
4	19	0.914	2007	Recent development	Mice model (0.30)
5	19	0.941	2015	Neuroinflammation regulation	Mice model (0.32)
6	16	0.979	2000	Following medial forebrain bundle axotomy	Mice model (0.22)
7	14	0.982	2009	Randomized controlled clinical trial	Mice model (0.08)
8	13	0.977	2011	Alternative medicine	Mice model (0.09)
9	13	0.938	2008	Microarray analysis	Mice model (0.13)
10	12	0.858	2015	Complementary therapy	Tetrahydropyridine-induced Parkinson’s disease model (0.09)
11	11	0.855	2011	Electromyographic recording	Mice model (0.26)
12	10	0.922	2004	Complementary acupuncture	Parkinson’s disease (0.08)
13	8	0.92	1998	Alternative therapy	Parkinson’s disease (0.13)

The cluster analysis results mainly include cluster ID, mean year, size, silhouette, label (LLR), and label (MI). Cluster ID is the number after clustering, and size represents the number of members contained in the cluster. The larger the size is, the smaller the number. The mean year represents the average year of the literature in the cluster, which can be used to judge the distance of the cited literature in the cluster. The larger the log-likelihood ratio (LLR), the more representative the cluster category; mutual information (MI) is mainly used to represent the relationship between terms and categories in text mining, and it does not consider the frequency of feature words.

## 4 Discussion

Based on the trend in annual publication output, it is possible to identify three distinct time periods. Initially, from 1995 to 2007, there was steady growth; followed by 2008–2014, which witnessed a slowdown in development; and finally, from 2015 to 2023, it reflects fluctuating trends. The pace and pattern of this development may be closely linked to the historical growth of acupuncture and moxibustion overseas, as well as China’s domestic policies for traditional Chinese medicine. Acupuncture entered Europe in the early 17th century. In 1821, British doctor John Churchill published a report on treating rheumatoid arthritis with acupuncture. In 1823, the first issue of the Lancet talked about acupuncture (Liguo et al., 2011). In the 1970s, the original acupuncture guild organization, the British acupuncture Registration Association, was established. Since the 1990s, the number of acupuncture practitioners has increased dramatically to 1995, and acupuncture practitioners across the UK have developed into the second largest complementary medicine in the whole complementary medicine field, which is only second to Western bone setting therapy. In July 2016, acupuncture received an independent professional code in the United States. It indicates that traditional Chinese medicine is accelerating the pace of discipline construction, medical system standardization, and higher education reform to gradually obtain the status of an independent discipline (Yang, 2017). Acupuncture is increasingly recognized by people all over the world. In 2008, the “Traditional Chinese Medicine Law” was included in the legislative plan of the Chinese National People’s Congress. On 3 February 2014, the International Organization for Standardization released information: The international standard for “Disposable Sterile Acupuncture Needles” formulated by Chinese experts was officially published, becoming the first published international standard for traditional Chinese medicine. The “Traditional Chinese Medicine Law of the People’s Republic of China” was passed on 25 December 2016 and came into effect on 1 July 2017 (Xie et al., 2023). The trend of the total number of publications has a particular relationship with the development history of acupuncture and Chinese domestic TCM policies. With the support of government policies, acupuncture, one of the crucial components of TCM, will naturally develop more rapidly. It can be attested by the development trend of cumulative publications, which manifests a year-by-year growth pattern and has evolved even more rapidly since 2008.

China, South Korea, and the USA are the leading countries researching acupuncture for Parkinson’s disease. The countries with the most fantastic cooperation are among the three, while the regions with the most germane collaboration are archly fastened on developed countries in Europe. Among the top 10 institutions, China and South Korea account for 50% of the total. The institutional cooperation is closer with Capital Medical University, Guangzhou University of Chinese Medicine, and Zhejiang Chinese Medical University as the core regional cooperation. Most of these cooperations are limited to Chinese universities of traditional Chinese medicine and with foreign countries. Institutional collaboration could be much better. As a consequence, we recommend that in the future, efforts should be made to strengthen cooperation between national institutions to promote the active development of acupuncture treatment for Parkinson’s disease.

Among the top 10 journals publishing the most studies on acupuncture for PD, Neurology boasts the highest impact factor at 10.1, regarded as the premier journal in this field. In addition, “Movement Disorders” ranks alongside “Brain Research” and “PLOS ONE” as top journals for scientific research output in this field. The most pivotal journal is Brain Research. These findings enable researchers to swiftly pinpoint the primary research outcomes in this domain. Moreover, when analyzing the frequency and centrality of journals, it is evident that the majority primarily concentrate on neuroscience and traditional Chinese medicine, with fewer being comprehensive journals. This will be a primary focus of our future endeavors. Moreover, the results from journal clustering analysis can rapidly aid scholars in pinpointing research themes within the field. Historically, numerous studies have been published on acupuncture in animal models for Parkinson’s disease and gene expression related to thalamic degeneration.

Park HJ is the most prolific author in the field of acupuncture for Parkinson’s disease, focusing on the neuroprotective mechanisms of acupuncture for PD. Yeo S belongs to South Korea, and his study concerns the neural response of acupuncture at Yang ling-quan acupoint to activate PD. At the same time, Cho SY is involved in clinical research on combining acupuncture and bee venom needle treatment for PD. They all come from South Korea, and through the author’s collaborative network, it can be seen that most scholars are limited to South Korea and lack cooperation with researchers from other countries. It is also a direction that should be pursued in the future, as seen through the Cited Author. The author’s cluster analysis indicates that the primary research focus of the researchers is on the MPTP-induced mouse model, tetrahydropyridine-induced mouse model, and a systematic review of randomized trials. The strongest citation burst detection is used to reflect the study plume within a certain time node, then through “Visualization,” and we can obtain the strongest citation burst detection results through the “Citation/Frequency Burst History” function. The red bar indicates the duration of the citation burst and the start and end years of the citation burst (Chen, 2018). The author who displays the most red squares in Figure 3D is Wang XM, so we believe he is a popular researcher. The hot author is Wang XM. Research has prompted that long-term high-frequency electroacupuncture stimulation of the Dazhui and Baihui acupoints can not only prevent the degeneration of dopaminergic neurons in the substantia nigra but also upregulate the level of BDNF mRNA in the ventral midbrain field, achieving the goal of improving PD (Liang et al., 2002). Liang et al. (2003) discovered that high-frequency electroacupuncture stimulation can upregulate the mRNA of glial cell-derived neurotrophic factor (GDNF) on both sides of the globus pallidus, which may be the reason for the effectiveness of electroacupuncture for PD. It can be seen that these scholars are core in the field of acupuncture for PD. Cited Author clustering analysis can reveal that research focuses on the basal ganglia output pathway, postmedial forebrain bundle transection, neuroprotective effects, cortical rearrangement, intestinal motility disorders, and systematic reviews. ① **Basal ganglia output pathway:** Electroacupuncture stimulation-induced unilateral transection of the medial forebrain bundle (MFB) in PD rats revealed that high-frequency stimulation can increase the normalization of GABA content in the basal ganglia output pathway, thereby improving motor disorders (Jia et al., 2010). ② **Neuroprotective effect:** Yoon et al. (2013) studied that bee venom acupuncture can quantitatively observe the neuroprotective effect of PD mouse models through immunohistochemistry and 1H-MRS. Doo et al. (2010) suggests that bee venom acupuncture can inhibit Jun activation to protect dopamine neurons from neurotoxicity, providing a new treatment strategy for PD. ③ **Cerebral Cortex Rearrangement:** Jang et al. (2020) probed the impact of acupuncture on Parkinson’s gait barrier, and this basis analyzed the blood flow dynamic influence of Parkinson’s patients with cerebral cortex. The results reveal that acupuncture tends to improve low-measurement gait and re-arrange the activation of the cerebral cortex. ④ **Intestinal Motility Disorder:** Electroacupuncture stimulation Thy1-αSyn transgenic PD mice with intestinal peristalsis disorder were treated at points Tianshu and Baihui, and the small intestine propulsion speed of PD mice was improved by intervening in serotonin levels, serotonin 4 receptor expression, and cAMP/PKA pathway, achieving the effect of improving constipation (Shen et al., 2022). ⑤ **System review:** The systematic review contains three main aspects, including targeted treatment methods [including abdominal acupuncture (Fang et al., 2022), scalp acupuncture (Lee et al., 2013), and combination of acupuncture and medicine (Liu et al., 2017)], evaluation of non-motor symptoms of Parkinson’s disease [including neurological and psychiatric symptoms (Zhang et al., 2024), sleep quality (Lin et al., 2024), fatigue (Zhi and Gao, 2020), lower urinary tract symptoms (Kim et al., 2018), and research on animal models (Lee et al., 2016)]. The commonality of these studies is that the research evidence is insufficient, and larger sample sizes of prospective, well-designed randomized controlled trials are still needed to further validate the conclusions. The hot topic Cited Author is Eisenberg DM. Through a questionnaire survey, data on the use of complementary and alternative therapies to treat anxiety and depression in the USA were observed. People with anxiety and depression, as well as patients with psychological problems, are increasingly using complementary and alternative therapies (Kessler et al., 2001).

Frequency analysis of keywords detected that “mouse model,” “messenger-RNA,” “systematic review,” and “nonmotor symptoms” have high frequencies, indicating that acupuncture is the main direction in treating non-motor symptoms of PD, among which both related to basic research on the mechanism, and systematic reviews of randomized controlled trials. The principal research contents involved in the keyword cluster analysis include microglial activation, massage therapy, MPTP-induced Parkinson’s disease mouse model, microbial dysbiosis, brain-gut peptide, basal ganglia output pathway, and MPTP-intoxicated mouse model. Keyword clustering involves the following research contents: ① **Microglial activation:** Bee venom acupuncture at Zu Sanli point in MPTP-induced Parkinson’s disease mice can weaken the activation of microglial responses and exert a neuroprotective effect (Kim et al., 2011). ② **Microbial dysbiosis:** Acupuncture in Zu Sanli and Yang Lingquan of PD mice not only increased the levels of dopaminergic fibers and neurons in the neostriatum but also restored the overexpression of microglia and astrocytes (Jang et al., 2020), thereby achieving the effect of inhibiting neuroinflammation. ③ **Brain-gut peptide:** Yu et al. (2020) explored that acupuncture at Zhong Wan, Qi hai, Zu Sanli, and Tai Chong acupoints in rats with non-motor symptoms of Parkinson’s disease can inhibit neuronal apoptosis and can also improve the rats’ spatial memory and weaken anxiety and depression, by regulating brain-gut peptide in rats to achieve the purpose of alleviating non-motor symptoms of PD. ④ **Massage therapy:** Ghaffari and Kluger (2014) and others reviewed common alternative therapies for Parkinson’s disease (including massage therapy). The results displayed that these treatments can reduce stress, improve mood and sleep and are indispensable in the treatment of Parkinson’s patients. ⑤ **MPTP-intoxicated mouse model:** Choi et al. (2011) identified that acupuncture in Yang Lingquan and Tai Chong of MPTP-induced Parkinson’s syndrome mice can inhibit the degeneration of nigrostriatal neurons to achieve a therapeutic effect. ⑥ **MPTP-induced Parkinson’s disease mouse model:** Kang et al. (2007) inspected that acupuncture at Yang Lingquan and Tai Chong acupoints in MPTP-induced Parkinson’s disease mice not only reduced MPTP-induced microglial activation and inflammation but also protected MPTP-induced damage to dopaminergic neurons, which can be assessed by changes in MAC-1, COX-2, and iNOS. Moreover, the burst detection analysis of keywords traced that the hot research keyword in this field is messenger RNA in the substantia nigra.

This study summarizes the Cited Reference of acupuncture for Parkinson’s disease as follows: The prominent document is Yeong Gon Choi’s research published in Neuroscience Letters in 2009 on “Acupuncture inhibits ferric iron deposition and ferritin-heavy chain reduction in an MPTP-induced parkinsonism model” (Choi et al., 2009), and acupuncture inhibited the decrease in tyrosine hydroxylase and dopamine transporter immunoreactivity caused by neurotoxicity and also inhibited the increase in iron and the decrease in ferritin-heavy chain, ultimately inhibiting the iron-related oxidative damage. The hot literature is the research article “Trends in alternative medicine use in the United States, 1990–1997: results of a follow-up national survey” published by Eisenberg DM in “JAMA-the Journal of the American Medical Association” in 1998 (Eisenberg et al., 1998). Eisenberg DM et al. studied the trend of alternative medicine utilized in the United States from 1990 to 1997. The substantial increase in the use and expenditure of alternative medicines was mainly due to the increase in the number of people seeking alternative therapies rather than the increase in the number of medical visits.

Cited Reference clustering research mainly includes the following parts: ① **Non-motor symptoms:** Xu et al. (2020) surveyed a multi-center RCT of madopar combined with acupuncture to treat Parkinson’s symptoms and non-motor symptoms and discovered that acupuncture and drug treatment can convert the symptoms at the same time. Improvements in motor and non-motor functions and non-motor symptoms (such as mental, cognitive, emotional, behavioral, and language) in patients with PD appear earlier than motor symptoms. ② **Neuroinflammation regulation:** Jang et al. (2020) observed that acupuncture treatment can improve motor function and anxiety in PD mice. Second, acupuncture treatment increased the levels of dopaminergic fibers and neurons in the nigrostriatal body to block the inflammatory response, and cell apoptosis to achieve therapeutic purposes. ③ **Randomized controlled trial:** Randomized controlled trials were conducted to observe the study of acupuncture on motor and non-motor symptoms of PD, mainly including constipation, anxiety, fatigue, and gait disorders. These are the predominant components of reference cluster analysis.

## 5 Conclusion

This study offers fresh insights into the trend of acupuncture treatments for Parkinson’s disease. Despite certain limitations, it offers a comprehensive analysis of the global trends in acupuncture for Parkinson’s disease, visualized in a knowledge graph for researchers. It reveals that China leads in scientific output within this field, with the strongest international collaborations occurring primarily in Europe. Inter-institutional collaborations are confined to higher education institutions specializing in traditional Chinese medicine. Park HJ stands as the most prolific author in this field, particularly Kim SN, the most significant Cited Author. Eisenberg DM is the most frequently cited author, and Movement Disorders is the journal with the most publications in this field. The key journal is Brain Research, with research hotspots on substantia nigra, messenger RNA, etc. The central cited reference is a 1998 paper by Eisenberg DM.

In conclusion, future studies will build upon large-scale RCT and cohort research to delve deeper into their specific clinical treatment benefits. In addition, the primary focus of future research will be the integration of acupuncture and pharmacological interventions for treating non-motor symptoms of Parkinson’s disease. In brief, the findings of this study could offer valuable insights for researchers.

## Data Availability

The original contributions presented in the study are included in the article/Supplementary material, further inquiries can be directed to the corresponding authors.
