# Supplementary figures and images for: Mapping knowledge domain of acupuncture for Parkinson’s disease: a bibliometric and visual analysis
Source: Front Aging Neurosci. 2024 Sep 4;16:1388290. doi: 10.3389/fnagi.2024.1388290 (PMC11408212; doi:10.3389/fnagi.2024.1388290)

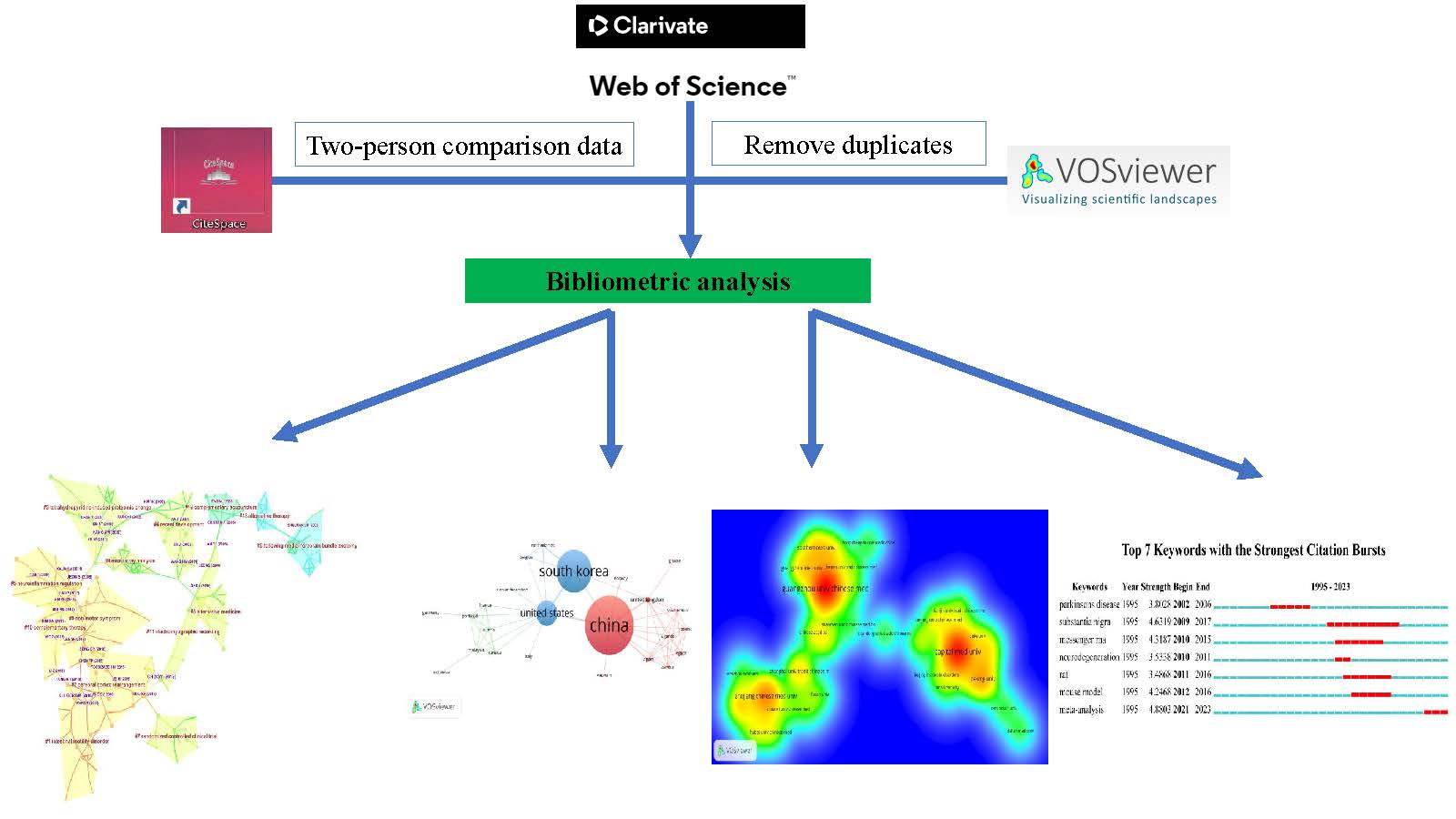

Supplement: Supplementary file 1 [file Image_8.jpg]
